# 3D cardiac shape analysis with variational point cloud autoencoders for myocardial infarction prediction and virtual heart synthesis

**DOI:** 10.1016/j.compmedimag.2025.102587

**Published:** 2025-06-19

**Authors:** Marcel Beetz, Abhirup Banerjee, Lei Li, Julia Camps, Blanca Rodriguez, Vicente Grau

**Affiliations:** a Institute of Biomedical Engineering, Department of Engineering Science, University of Oxford, Oxford OX3 7DQ, United Kingdom; b Division of Cardiovascular Medicine, Radcliffe Department of Medicine, University of Oxford, Oxford OX3 9DU, United Kingdom; c Department of Computer Science, University of Oxford, Oxford OX1 3QD, United Kingdom

**Keywords:** 3D anatomy modeling, Cardiac electrophysiology simulation, Geometric deep learning, Myocardial infarction prediction, Point cloud VAE, Virtual population generation

## Abstract

Cardiac anatomy and physiology vary considerably across the human population. Understanding and taking into account this variability is crucial for both accurate clinical decision-making and realistic *in silico* modeling of cardiac function. In this work, we propose multi-class variational point cloud autoencoders (Point VAE) as a novel geometric deep learning approach for 3D cardiac shape and function analysis. Its encoder–decoder architecture enables efficient multi-scale feature learning directly on high resolution point cloud representations of the multi-class 3D cardiac anatomy and can capture complex non-linear 3D shape variability in a low-dimensional and interpretable latent space. We first evaluate the Point VAE’s reconstruction ability on a dataset of over 10,000 subjects and find mean Chamfer distances between input and reconstructed point clouds below the pixel resolution of the underlying image acquisitions. Furthermore, we analyze the Point VAE’s latent space and observe a realistic and disentangled representation of morphological and functional variability. We test the latent space for pathology prediction and find it to outperform clinical benchmarks by 13% and 16% in area under the receiver operating characteristic (AUROC) curves for the tasks of prevalent myocardial infarction (MI) detection and incident MI prediction, respectively, and by 10% in terms of Harrell’s concordance index for MI survival analysis. Finally, we use the generated populations for *in silico* simulations of cardiac electrophysiology, demonstrating its ability to introduce realistic natural variability.

## 1 Introduction

Cardiac anatomy and physiology vary considerably across the human population. Understanding and taking into account this variability is of key importance for accurate clinical decision-making and for the development of realistic computational models that faithfully reflect true cardiac shape and function (Young and Frangi, 2009; Fonseca et al., 2011). It is also a key concept behind the desirable goal of personalized medicine and the cardiac digital twin, which promise to considerably improve the diagnosis and treatment of cardiovascular disease, the most common cause of death in the world (Young and Frangi, 2009; Fonseca et al., 2011; Corral-Acero et al., 2020).

Accordingly, considerable research has been devoted to the analysis of cardiac shape and function, including the incorporation of subject-specific differences. The most commonly used statistical shape modeling technique for cardiac analysis is principal component analysis (PCA), as it allows for the straightforward and interpretable extraction of the most important modes of shape variation (Bai et al., 2015; Ardekani et al., 2016; Farrar et al., 2016; Gilbert et al., 2019; Mauger et al., 2019; Gilbert et al., 2020; Corral-Acero et al., 2020; Mauger et al., 2021; Rodero et al., 2021; Romero et al., 2021). However, PCA captures only linear relationships, does not necessarily preserve local information in the transformed feature space, is sensitive to outlier and missing values, and relies on a simplified mathematical framework which limits modeling capabilities. Furthermore, it typically requires high-quality mesh representations of the 3D cardiac anatomy with vertex connectivity shared across the dataset, whose acquisition from clinical data is difficult and time-consuming.

More recently, deep learning techniques have become more prevalent in 3D heart modeling (Gilbert et al., 2020; Puyol-Antón et al., 2020). Compared to traditional statistical methods, deep learning networks typically exhibit a more complex structure with a larger number of trainable parameters and extensive use of non-linearities and thus promise to be able to model more comprehensive variability patterns. In particular the variational autoencoder (VAE) (Kingma and Welling, 2013) has been increasingly used to model cardiac shape variability. Its advantages include computational efficiency, ease and stability of training, and a latent space representation that facilitates model interpretability and thus clinical acceptance. This sets it apart from other generative deep learning models, such as generative adversarial networks. However, in order to apply typical deep learning operations, such as convolutions, previous approaches use regular grid structures (e.g. images, voxelgrids) to store highly sparse cardiac surface data, which is considerably less efficient in terms of time and memory compared to geometric representations of the same underlying anatomy such as point clouds or meshes.

In order to overcome these drawbacks, geometric deep learning (Bronstein et al., 2017) techniques have recently been developed to allow for the applications of deep learning operations directly on non-Euclidean data. For example, neural networks capable of processing mesh representations of cardiac shape have been proposed for a variety of tasks related to cardiac shape analysis, including 3D shape reconstruction (Kong et al., 2021; Beetz et al., 2022b), generation of virtual heart populations (Beetz et al., 2022d; Dou et al., 2022), simulation of cardiac mechanics (Dalton et al., 2021) and motion (Lu et al., 2020, 2021a,b; Meng et al., 2022; Beetz et al., 2023), outcome prediction (Beetz et al., 2023e), wall shear stress estimation (Suk et al., 2021), prediction of depolarization times (Meister et al., 2020), and cardiac anatomy modeling (Beetz et al., 2022d).

However, mesh-based deep learning approaches typically rely on multiple assumptions, such as the existence of shared vertex correspondence across the dataset, which has to be established in potentially complicated and error-prone pre-processing steps, often based on pre-selected template meshes. On the other hand, point cloud-based deep learning offers an alternative, more flexible way to process the same 3D cardiac shapes in the form of point clouds which are easier to acquire and do not rely on explicit connectivity encodings or vertex-to-vertex correspondence to be present in the data. This considerably facilitates data preparation pipelines and improves its applicability in a variety of settings. In addition, point clouds encode 3D shapes as a simple list of x,y,z coordinates making them easier to manage while allowing for the straightforward inclusion of class information as additional feature values, for example to identify different anatomical substructures. This data format also enables point clouds to represent complex 3D shapes while retaining high efficiency when storing anatomical information, reducing memory requirements, enabling higher data resolutions, and improving accuracy. Accordingly, point cloud deep learning has seen applications in various cardiac shape analysis settings, such as disease detection (Chang and Jung, 2020; Beetz et al., 2023a), joint left ventricular (LV) wall segmentation and reconstruction (Ye et al., 2020), vessel classification and segmentation (Yu et al., 2021), probabilistic motion (Zakeri et al., 2022) and deformation (Beetz et al., 2021c; Seale et al., 2024) modeling, 3D shape reconstruction (Beetz et al., 2021a, 2023b; Xiong et al., 2022), and combined cardiac shape and electrocardiogram modeling (Beetz et al., 2022a,c; Li et al., 2023).

We present the multi-class variational point cloud autoencoder (Point VAE) as a novel geometric deep learning approach to model population-wide variability in 3D cardiac morphology and function. Its encoder–decoder architecture is specifically designed to directly and simultaneously process point cloud representations of multiple cardiac substructures at multiple phases of the cardiac cycle in a single unified model. This allows the network to efficiently capture complex non-linear population-wide variability in both cardiac anatomy and function in a low-dimensional and general-purpose latent space, maintaining a high degree of interpretability. Different from task-specific deep learning approaches which are limited to a single application, the Point VAE captures general 3D patterns that are suitable for many downstream tasks, such as pathology prediction and virtual heart generation. It achieves this without requiring any point correspondence like previous statistical shape models (Corral Acero et al., 2022) or mesh-based deep learning techniques (Beetz et al., 2022d) and thus exhibits a high degree of flexibility and applicability to act as a key component in a fast and fully automatic 3D cardiac shape modeling pipeline.

We showcase the utility and versatility of our approach for two important down-stream use cases. First, we show the ability of the Point VAE’s latent space representation to outperform multiple clinical benchmarks in the tasks of prevalent myocardial infarction (MI) detection, incident MI prediction, and MI survival analysis. 3D shape biomarkers are known to play a major role in the understanding and diagnosis of MI (Suinesiaputra et al., 2017; Baessler et al., 2018; Cetin et al., 2018; Isensee et al., 2018; Khened et al., 2018; Wolterink et al., 2018; Corral Acero et al., 2022), making it an ideal direction for our approach. Second, we demonstrate the Point VAE’s ability to generate realistic virtual populations of 3D anatomies with similar clinical metrics as the true population. This way, the Point VAE can act as a data augmentation method in a variety of down-stream research and clinical applications (e.g. cardiovascular disease classification). We specifically demonstrate the utility of the generated population as inputs for *in silico* simulations of cardiac electrophysiology giving them access to a large amount of additional population variability in an efficient manner. This improves modeling quality, and offers the potential to reduce the number of required animal trials and accelerate the discovery and validation of novel medical devices and drugs.

In summary, we make the following main contributions:

- We develop the Point VAE as a novel geometric deep learning approach to efficiently analyze 3D cardiac anatomy and function based on high-resolution point cloud representations automatically reconstructed from gold standard cardiac MR acquisitions;
- We evaluate the Point VAE’s ability to accurately encode and reconstruct 3D cardiac shapes in a combined multi-temporal and multi-class setting on a large UK Biobank dataset and analyze its latent space as a low-dimensional and interpretable representation of 3D heart variability;
- We study the suitability of the learned latent space representation for multiple MI classification tasks and demonstrate its outperformance relative to current clinical gold standards; and
- We assess the Point VAE’s capability to generate realistic populations of 3D hearts and investigate their suitability for electrophysiology simulations by proposing a novel fully-automatic post-processing pipeline.

## 2 Methods

In this section, we first give an overview of the proposed method (Section 2.1), before describing the dataset (Section 2.2), preprocessing steps (Section 2.3), network architecture (Section 2.4), training procedure (Section 2.5), and sample use cases (Sections 2.6–2.7) in greater detail.

### 2.1 Overview

**Fig. 1:**
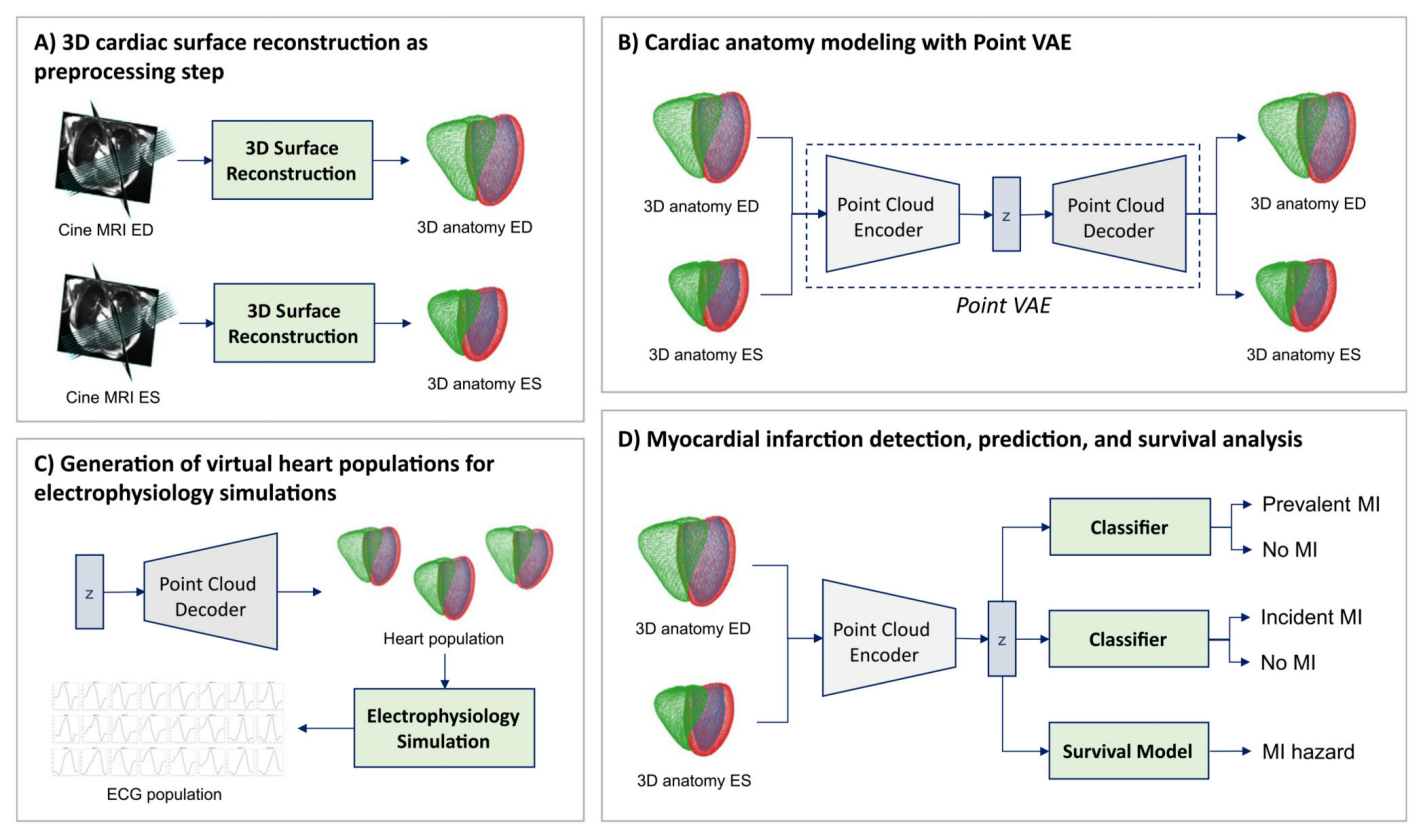
Overview of the proposed cardiac anatomy modeling pipeline. We first reconstruct multi-class point clouds to represent the anatomical surfaces of three cardiac substructures (LV endocardium, LV epicardium, right ventricular (RV) endocardium) at both the ED and ES phases using a multi-step automated pipeline (A). The ED and ES point clouds are then concatenated and used to train a multi-class Point VAE to capture 3D cardiac shape variability in a low-dimensional latent space *z* (B). We then investigate the utility of this compact representation *z* for four applications, namely, prevalent MI detection, incident MI prediction, MI survival analysis, and the generation of virtual population cohorts for *in silico* simulations of cardiac electrophysiology (C,D).

We propose a novel geometric deep learning-based pipeline with a variational point cloud autoencoder as a key component for efficient 3D cardiac shape and function modeling and showcase its suitability for multiple downstream tasks (Fig. 1).

Given raw cine MRI acquisitions, we first reconstruct 3D cardiac anatomies in the form of multi-class point clouds at both the end-diastolic (ED) and end-systolic (ES) phases of the cardiac cycle using a multi-step deep learning approach (Fig. 1-A; Section 2.3). We then use the obtained point clouds as inputs to a Point VAE to capture population-wide variability in 3D cardiac shapes in a low-dimensional latent space representation (Fig. 1-B; Section 2.4). We train two separate networks, one on ED data only and one on combined ED and ES data, to investigate the Point VAE’s ability to learn both uni-temporal and multi-temporal 3D shape and function patterns. The pre-trained Point VAE is then used for multiple follow-up tasks to showcase its versatility. First, we develop a machine learning classifier based on the PointVAE’s latent space to detect prevalent MI, predict incident MI, and perform MI survival analysis (Fig. 1-D; Section 3.3). Next, we generate realistic virtual heart populations and use them as inputs to run *in silico* simulations of cardiac electrophysiology (Fig. 1-C; Section 2.7).

### 2.2 Dataset

Our dataset consists of 10,237 subjects of the UK Biobank study (Petersen et al., 2013). For each case, cine magnetic resonance images were acquired using a balanced steady-state free precession (bSSFP) protocol with a consistent voxel resolution of 1.8 × 1.8 × 8.0 mm^3^ for short-axis images and 1.8 × 1.8 × 6.0 mm^3^ for long-axis images (Petersen et al., 2015). Based on UK Biobank field ID 42000 (Date of myocardial infarction), 294 subjects had a myocardial infarction event prior to image acquisition (prevalent MI), while 235 subjects suffered an infarction event after imaging (incident MI). Among the remaining subjects, we identified 4864 subjects with some form of cardiovascular disease or other common pathologies, which we define following the selection criteria in previous studies (Bai et al., 2020; Beetz et al., 2022a) (see Table 2 in the Appendix). The remaining 5373 subjects are considered as the control group in this work.

### 2.3 Preprocessing

The raw cine MR images form the basis of our proposed 3D shape modeling pipeline. In order to obtain 3D surface representations of the cardiac anatomy from a set of intersecting 2D MRI slices, we apply the multi-step deep learning-based reconstruction pipeline described in Beetz et al. (2023c, 2021a). It starts by segmenting the short-axis and four-chamber long-axis MRI slices into four anatomical regions, namely LV bloodpool, LV myocardium, right ventricular (RV) blood-pool, and background, with a pre-trained fully convolutional neural network (Bai et al., 2018) and the two-chamber long-axis MRI slices into three anatomical regions, namely LV bloodpool, LV myocardium, and background, with a U-Net pre-trained with adversarial training. The contour lines of the segmented anatomical regions, namely LV endocardium, LV epicardium, and RV endocardium, are then extracted from the segmentation masks of each slice and placed into 3D space as a labeled point cloud. The coordinate system of the point cloud is then adjusted via a rigid transformation to approximately match the one used during the training process of the deep learning reconstruction network. Finally, a multi-class point cloud completion network converts the 3D contour points into a dense surface representation of the cardiac anatomy, which serves as the input for the subsequent anatomy modeling step. We apply the reconstruction process separately to the slices at both the ED and ES phases of the cardiac cycle of each subject. Hereby, we select the first frame of the associated cine MRI sequence as the ED phase and the frame with the smallest volume in its segmentation mask as the ES phase (Banerjee et al., 2021). More detail about the data preprocessing steps can be found in Beetz et al. (2023c).

### 2.4 Point VAE architecture

**Fig. 2:**
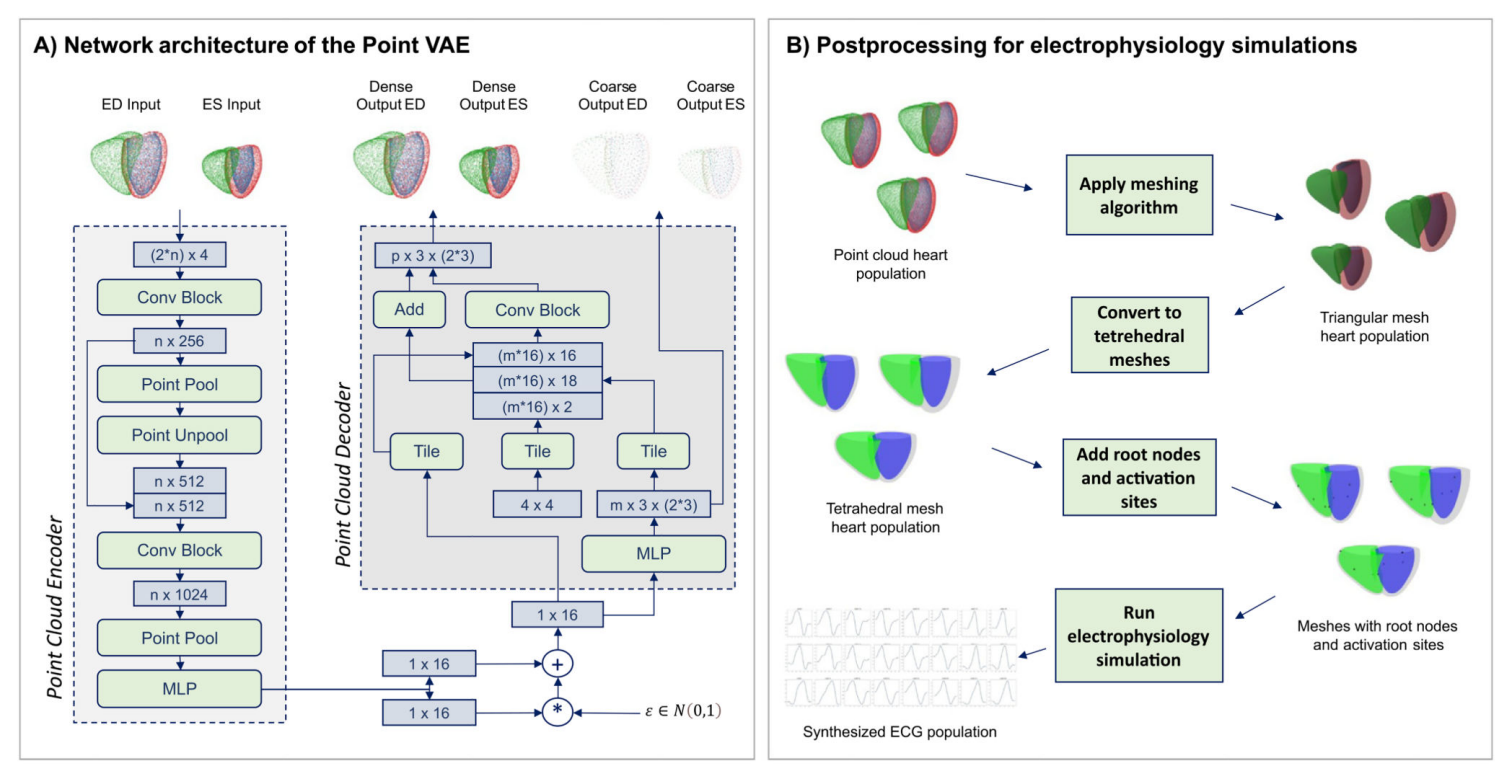
Overview of the encoder–decoder architecture of the proposed multi-class Point VAE (A) and the post-processing pipeline for cardiac electrophysiology simulations (B). (A) The encoder of the Point VAE consists of two stacked PointNet-style (Qi et al., 2017a,b) blocks with a multi-layer perceptron (MLP) to enable direct processing of multi-class point clouds of size (2 * *n*) × 4, where 2 refers to the ED and ES phases, *n* to the number of points, and 4 to the spatial x,y,z coordinates and the class label defining the respective anatomical substructure. The decoder combines a MLP with a FoldingNet-style (Yang et al., 2017) structure to output both a coarse low-resolution point cloud (*m* × 3 × (2 * 3)), which facilitates global feature learning during training, and a dense high-resolution point cloud (*p* × 3 × (2 * 3)), which acts as the network’s final prediction for the reconstructed anatomy. Hereby, *m* and *p* refer to the respective number of points with *m* ≪ *p*, 3 to the three coordinates x,y,z, and 2 * 3 to the three cardiac substructures at the ED and ES phases. Encoder and decoder are connected by a low-dimensional latent space vector. (B) To prepare generated data for *in silico* trials, point clouds are first converted to triangular meshes and then remeshed for tetrahedralization. The locations of earliest activation sites are set to seven fixed homologous locations, before performing the final electrophysiology simulations.

The architecture of the Point VAE follows an encoder–decoder structure with point cloud-based deep learning components (Qi et al., 2017a,b; Yang et al., 2017; Yuan et al., 2018) embedded in a variational autoencoder (Kingma and Welling, 2013) framework (Fig. 2-A).

It receives 3D multi-class point clouds encoded as (2 * *n*) × 4 tensors as inputs, where 2 refers to the ED and ES phase, *n* to the number of input points, and 4 to the x,y,z coordinates and class label. We represent the biventricular anatomy as three substructures with class labels 1, 2, and 3 referring to the LV endocardium, LV epicardium, and RV endocardium, respectively. For combined modeling of ED and ES data, we concatenate the two point clouds along the first tensor axis before feeding the result into the network. The network encoder is comprised of two PointNet-style (Qi et al., 2017a,b) blocks with a residual connection to enable direct and effective feature learning on point clouds followed by a multi-layer perceptron. It outputs two 16-dimensional vectors that represent the mean and standard deviation (SD) values of the multivariate latent space distribution. These are combined with a multivariate standard normal distribution as part of the reparameterization trick (Kingma and Welling, 2013) to enable both random sampling and gradient flow for network training. The resulting latent space vector is passed to the decoder, which is made up of two main parts. First, a multi-layer perceptron aims to reconstruct a low-resolution multi-class point cloud representation of the original input anatomy with size *m* × 3 × (2 * 3). Here, *m* refers to the number of points in the output point cloud, 3 to the x, y, z coordinates, and 2 * 3 to the three cardiac substructures at the ED and ES phases. In case of combined ED and ES outputs, a tensor with twice the size is used. The second part of the decoder follows a FoldingNet-style (Yang et al., 2017) architecture, where a 4 × 4 patch of points is placed around each point in the low-dimensional output of the previous step and then deformed via a combination of convolution, concatenation, and addition layers. The result is a high-resolution multi-class point cloud to provide a dense surface representation of the cardiac anatomy as the final network output. The point cloud is encoded in a similar way to the low-dimensional point cloud as a *p* × 3 × (2 * 3) tensor but with *p* ≫ *m*, as modulated by the FoldingNet step.

### 2.5 Loss function and training

Following the beta-VAE framework (Higgins et al., 2017; Kingma and Welling, 2013), the loss function of the Point VAE consists of two parts, a reconstruction loss term and the Kullback–Leibler (KL) divergence term, weighted by a parameter *β*.

(1) $$L_{total\;}=L_{reconstruction\;}+\beta *L_{KL}.$$

Depending on the value of *β*, the network puts more emphasis on producing accurate anatomy reconstructions or on obtaining a high latent space quality during training. In this work, we empirically select a *β* value of 0.2 as a reasonable trade-off between the two objectives given our particular dataset.

The KL divergence terms between the prior distribution *P* (*z*) and the posterior distribution *Q*(*z* | *X*) of the Point VAE’s latent space is defined as follows:

(2) $$L_{KL}=D_{KL}[Q(z|X)\|P(z)].$$

Here, *X* refers to the input anatomies and *z* to the latent space. The reconstruction loss term consists of two terms weighted by a parameter *α*.

(3) $$L_{reconstruction\;}=\overset{T}{\underset{i=1}{\sum }}\overset{C}{\underset{j=1}{\sum }}\left(L_{coarse,i,j}+\alpha *L_{dense,i,j}\right),$$

where *C* refers to the number of anatomical substructures encoded as classes and *T* to the number of cardiac phases. While we use values of 3 for *C* and both 1 and 2 for *T* in this work, the loss function can be easily extended to incorporate additional substructures and time points along the cardiac cycle. The coarse and dense loss terms compare the low-resolution and high-resolution point cloud outputs respectively to the input point cloud. We employ a monotonic annealing schedule (Bowman et al., 2015) for the weighting parameter *α* by setting it to lower values at the start of training to focus on an accurate reconstruction on a global level and then steadily increasing it as training progresses to also improve the reconstruction on a local level.

We select the symmetric Chamfer distance (CD) between the reconstructed point cloud *P*_1_ and input point cloud *P*_2_ for both the coarse and dense loss terms, as a widely used approximate surface distance between point clouds.

(4) $$CD\left(P_{1},P_{2}\right)=\frac{1}{2}\left(\frac{1}{\left|P_{1}\right|}\underset{x\in P_{1}}{\sum }\underset{y\in P_{2}}{\min}\|x-y{\|}_{2}+\frac{1}{\left|P_{2}\right|}\underset{y\in P_{2}}{\sum }\underset{x\in P_{1}}{\min}\|y-x{\|}_{2}\right).$$

We train our networks using the Adam (Kingma and Ba, 2015) optimizer with a batch size of 4 on a GeForce RTX 2070 Graphics Card with 8 GB memory. We apply a train/validation/test split of 70%/5%/25% to our dataset and select a training duration of 300,000 steps based on empirically observed loss convergence behavior on the validation dataset. The code is implemented using the TensorFlow (Abadi et al., 2016) and Scikit-learn (Pedregosa et al., 2011) libraries, respectively.

### 2.6 Survival prediction of myocardial infarction events

To prepare our dataset for the MI survival prediction task, we define January 1, 2021 as our study endpoint and consider all subjects without an MI event before this date to be right-censored. Next, we calculate the time between each subject’s imaging date and either the incident MI or censoring event as the survival/censoring time. We then use the obtained values to construct a hazard function and train a Cox regression model under the proportional hazard assumption (Cox, 1972).

(5) $$log\frac{h_{i}(t)}{h_{0}(t)}=\beta_{1}x_{i1}+\beta_{2}x_{i2}+\cdots +\beta_{p}x_{ip}.$$

Here, *h*_0_(*t*) refers to the baseline hazard, *h*_*i*_(*t*) to the predicted hazard, *β* to the regression coefficients, and *x* to the independent variables. For a quantitative model comparison, we select Harrell’s concordance index (Harrell et al., 1982) as our evaluation metric, defined as follows:

(6) $$C=\frac{\Sigma_{i,j}\delta_{i}\times I\left(\eta_{i}>\eta_{j}\right)\times I\left(t_{i}<t_{j}\right)}{\Sigma_{i,j}\delta_{i}\times I\left(t_{i}<t_{j}\right)}$$

where *δ* refers to the censoring state of a given subject (MI event or censored), *I*(·) to the binary indicator function, *η* to the survival risk predicted by the model, and *t* to the event time. The index provides a normalized score between 0 and 1 with an intuitive interpretation similar to AUROC, i.e., a value of 1 indicates perfect survival prediction by the model and a value of 0.5 indicates no differentiation.

### 2.7 Postprocessing for cardiac electrophysiology simulations

In order to run *in silico* simulations of cardiac electrophysiology on the point cloud anatomies in our dataset, we apply several post-processing steps (Fig. 2-B).

First, we convert the 3D point clouds of each anatomical substructure into triangular meshes with the Ball Pivoting algorithm (Bernardini et al., 1999) and automatically remove non two-manifold components. Since the RV epicardium cannot be resolved at the cine MR resolution, we warp the RV endocardial mesh surface outward along the direction of each vertex normal by 3 mm (Prakash, 1978) to produce an approximation of the RV epicardium. Then, we merge the LV and RV epicardial surfaces into a single biventricular epicardium by connecting the vertices of each ventricle which are closest to the LV-RV intersection curve.

Next, we join the generated LV endocardium, RV endocardium, and biventricular epicardium surfaces together at the basal plane to generate a watertight biventricular mesh, which is then remeshed with a restricted Frontal-Delaunay algorithm using the mesh generator JIGSAW (Engwirda, 2014) with an element size of 1.5 mm. We use TetGen (Si, 2015) on the remeshed 3D surface to build the final tetrahedral mesh, with the same 1.5 mm element size. For estimating the ECG electrode locations, we employ the automated 3D torso reconstruction pipeline presented in Smith et al. (2022, 2024).

To simulate ECGs, we employ an efficient Eikonal model (Camps et al., 2021) defined as follows:

(7) $$\sqrt{\left(\nabla d^{T}\cdot \nabla d\right)}=1.$$

Here, ∇*d* = *v*∇*t*, where ∇*t* is the traveling time passing through a node and *v* denotes the conduction velocities (CVs) of the fiber, transmural, and normal directions. The CVs along the fiber, transmural, normal, and endocardial directions are set to 67 cm/s, 30 cm/s, 17 cm/s, and 120 cm/s, respectively, to correspond to the reported velocities of the healthy ventricular myocardium (Caldwell et al., 2009; Mincholé et al., 2019). The locations of earliest activation sites are set to seven fixed homologous locations for realistic application (Cardone-Noott et al., 2016). Specifically, we select four earliest activation sites on the LV (LV mid septum, LV basal anterior paraseptal, and two LV mid-posterior) and three on the RV (RV mid-septum, two RV free wall) for each mesh. Note that here we only consider eight independent leads, as lead III and the augmented leads (aVF, aVR, and aVL) are linear combinations of other leads.

## 3 Experimental analysis

In this section, we first experimentally analyze the Point VAE’s reconstruction ability (Section 3.1) and latent space quality (Section 3.2) before investigating its utility for multiple sample applications, namely, MI detection, prediction, and survival analysis (Section 3.3), and the generation of virtual heart populations for cardiac electrophysiology simulations (Section 3.4).

### 3.1 Reconstruction ability

**Fig. 3:**
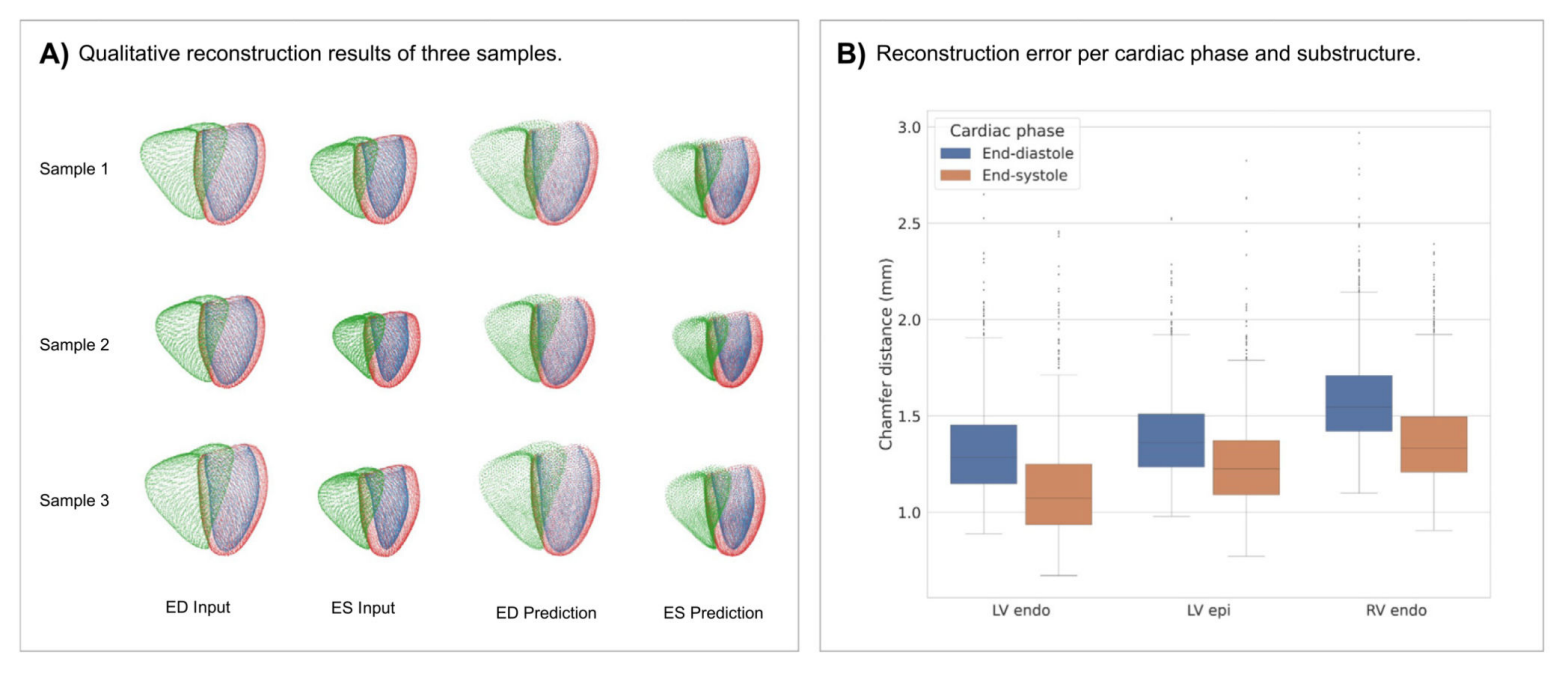
Qualitative and quantitative results of the Point VAE in the anatomy reconstruction experiments. (A) Input and reconstructed point clouds of three sample cases. (B) Reconstruction error of the Point VAE in terms of Chamfer distance between input and reconstruction, separated by cardiac substructures and phases.

The Point VAE is designed to be able to encode and reconstruct a variety of different multi-class input anatomies in a multi-temporal setting. To evaluate its suitability for this purpose, we select a large dataset of cine magnetic resonance images of over 10,000 subjects from the UK Biobank study (see Section 2.2 for more details). For each subject, we reconstruct the 3D surface representation of the biventricular anatomy consisting of three cardiac substructures, namely LV endocardium, LV epicardium, right ventricular (RV) endocardium, at the end-diastolic (ED) and end-systolic (ES) phases of the cardiac cycle (see Section 2.3 for more details). We then train the Point VAE to reconstruct the input ED and ES anatomies simultaneously while maintaining a disentangled and low-dimensional latent space (see Section 2.5 for more details). We then assess its performance on the unseen test dataset and depict the input point clouds and the corresponding reconstructed point clouds of three sample cases in Fig. 3-A.

We observe a high degree of alignment between the corresponding input and reconstructed shapes on both local and global levels in all sample cases. Cardiac substructure information is accurately retained in the reconstructed point clouds, which also show a similar point density distribution of their surface representation as the input point clouds. Typical changes between ED and ES anatomies, such as differences in volume or myocardial thickness, are also successfully preserved. To quantify the Point VAE’s reconstruction ability, we pass all point clouds of the unseen test dataset through the trained Point VAE and calculate the Chamfer distance between the input and reconstructed point clouds (Fig. 3-B). We find mean Chamfer distances lower than the voxel resolution of the underlying images (1.8 × 1.8 × 8.0 mm^3^) for all cardiac substructures and phases.

### 3.2 Latent space analysis

**Fig. 4:**
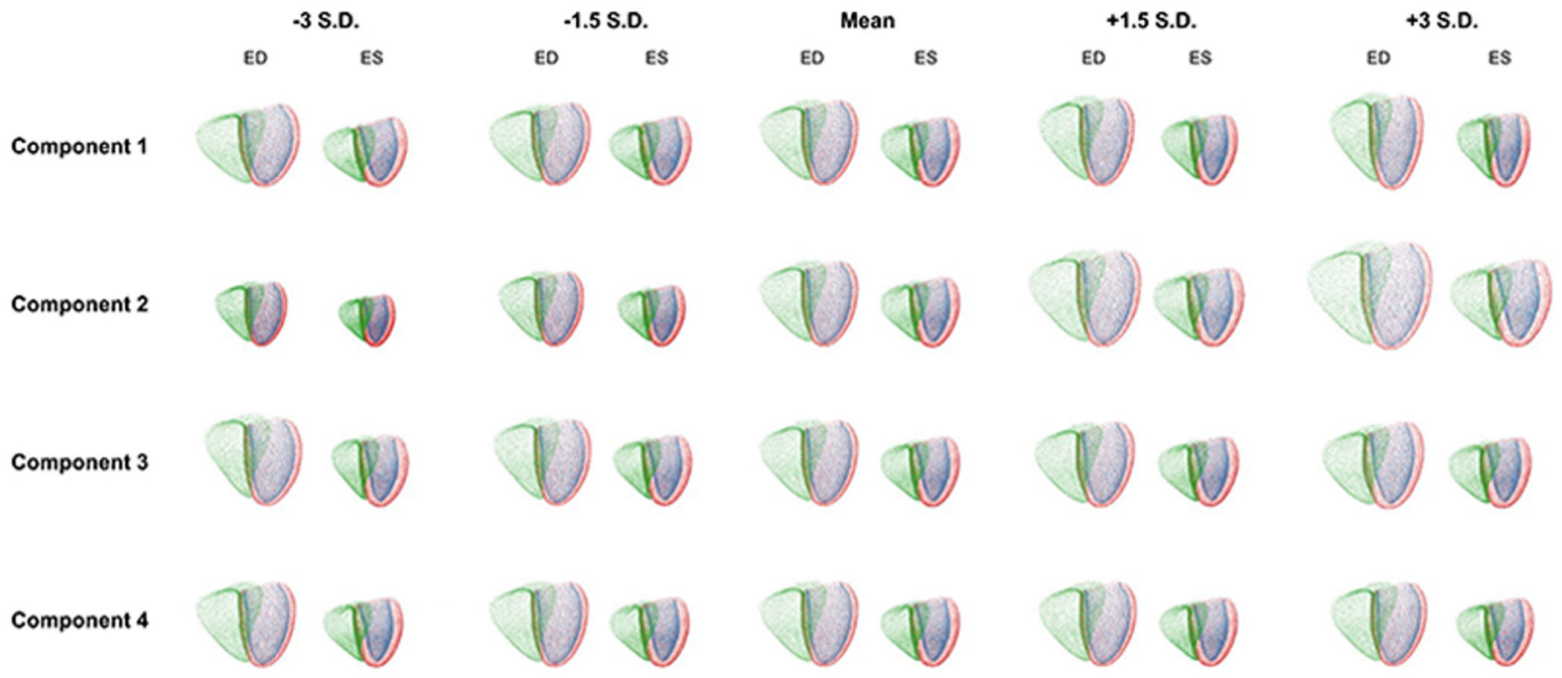
Effects of variations in four latent space components of the Point VAE on the reconstructed ED and ES anatomies.

The Point VAE’s latent space is a key advantage for cardiac shape modeling, as it provides a disentangled low-dimensional representation of the complex 3D shape variability which can be used to better understand the encoded shape phenotypes. As such, we further investigate its composition by analyzing the contributions of each component to different aspects of said shape variations. To this end, we vary individual component values in both the positive and negative directions while keeping the rest of the latent space at their mean values and feed the resulting vectors through the pre-trained decoder of the Point VAE. The shape changes in the resulting point cloud reconstructions can then be attributed to the variations in the corresponding latent space components. We depict three components with noticeable effects on the reconstructed shapes and one component with very little contribution for the Point VAE in Fig. 4.

For the contributing components, we observe easily identifiable shape changes in the reconstructed anatomies that mimic known aspects of population-wide shape variability. For example, component 1 is involved in modulating the basal plane tilt, component 2 the ventricular elongation, and component 3 the overall heart size. The changes appear gradually as the component is varied from negative to positive values. On the other hand, some components, such as component 4, have only a very small effect on the reconstructed shapes. On an individual level, all point clouds exhibit realistic shapes and maintain plausible relations between anatomical substructures. Furthermore, the ED and ES shapes undergo corresponding shape changes for each component. For example, the gradual increases in overall heart size when moving along component 2 from negative to positive values occur to a similar extent for both ED and ES shapes. As a consequence, the clinically known relationships between ED and ES shapes, such as a higher myocardial thickness at the ES phase, are maintained in a realistic way throughout all component variations.

### 3.3 Myocardial infarction classification

As 3D cardiac shape and function differences are known to be highly useful in cardiac disease diagnosis, we next analyze the utility of the latent space for the task of myocardial infarction prediction. To this end, we first pass an equal number of normal and MI cases from the unseen test dataset through the encoder of the Point VAE and extract the latent space vector for each case. We then train a logistic regression model with the individual components of the latent space as independent variables, for the task of binary classification into normal and MI subjects. For comparison, we select ejection fraction (EF) as the gold standard clinical metric for MI-related cardiac function assessment and use it as the independent variable to train three separate logistic regression models of LV EF only, RV EF only, and combined LV EF and RV EF, on the same dataset. We repeat this procedure separately for the tasks for prevalent MI detection and incident MI prediction and evaluate method performance with five common binary classification metrics, namely, Accuracy (Acc.), area under the receiver operating characteristic curve (AUROC), F1-score, Precision (Prec.), and Recall (Rec.). The average results of 10-fold cross validation experiments are shown in columns 2 to 11 in Table 1.

We find that the Point VAE’s latent space outperforms the respective clinical benchmarks for all evaluation metrics and both classification tasks. Specifically, it improves AUROC scores by 16% and 13% for the tasks of prevalent MI detection and incident MI prediction, respectively. Scores are generally higher for the task of prevalent MI classification. Next, we extend the binary MI classification objective to survival analysis with MI outcomes and evaluate the utility of the Point VAE’s latent space components as independent variables in a Cox regression model (Cox, 1972) (see Section 2.6 for more details). We also train additional regression models with LV EF, RV EF, and combined LV and RV EF as independent variables, as the clinical benchmark approaches and report the average Harrell’s concordance index scores (Harrell et al., 1982) of 10-fold cross validation experiments in the rightmost column of Table 1. The regression model with latent space inputs achieves higher scores as compared to all clinical benchmarks, with an improvement of about 10% over the best performing baseline.

### 3.4 Virtual population generation

**Fig. 5:**
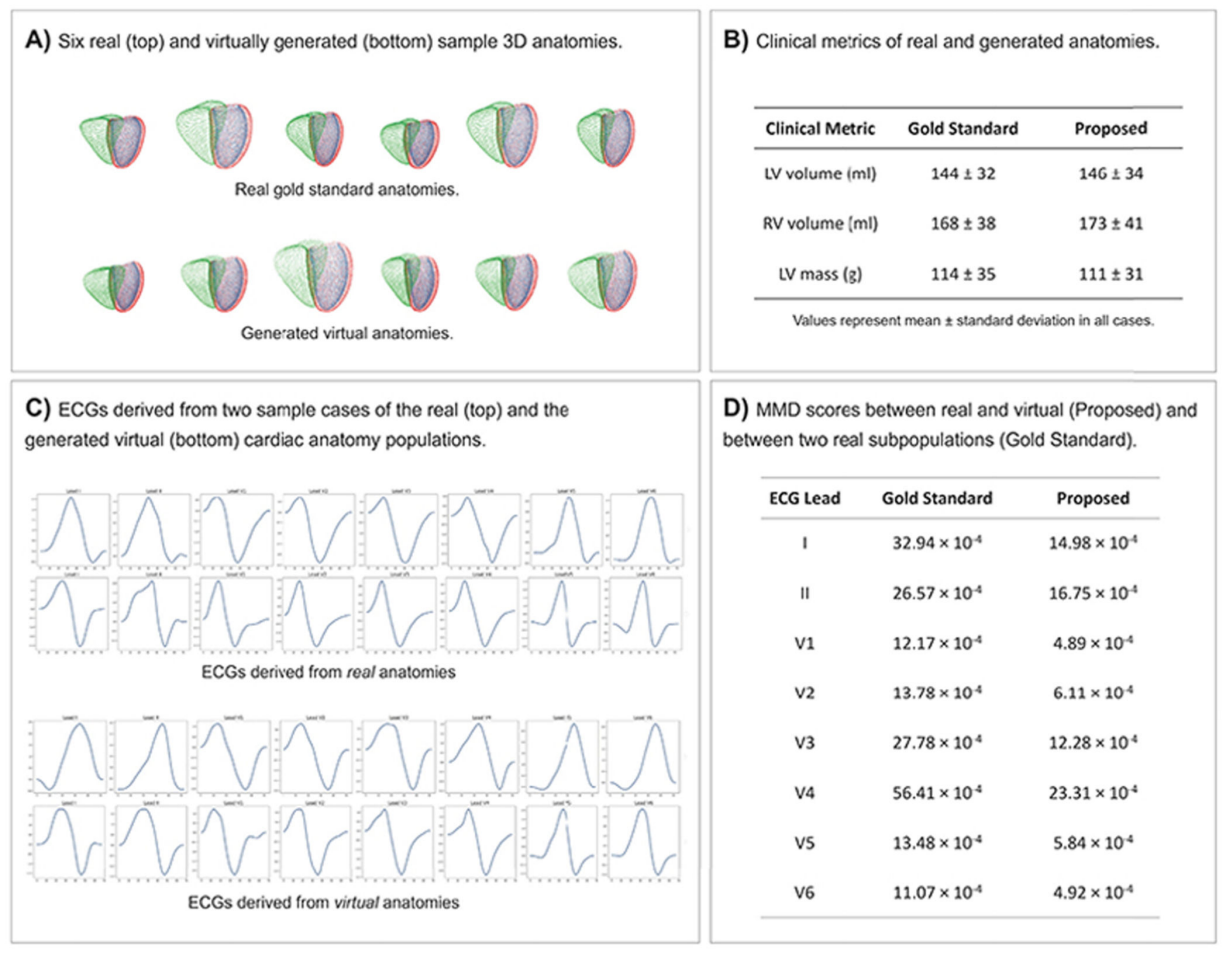
Qualitative and quantitative results of the experiments on virtual population generation (A,B) and *in silico* simulations of cardiac electrophysiology (C,D). (A) Six sample anatomies from the real population (top) and from the virtual population generated by the Point VAE (bottom). (B) Clinical metrics of LV volume, RV volume, and LV mass of the real and virtual generated anatomies. (C) ECGs derived from two sample cases (rows) of the real (top) and the generated virtual (bottom) cardiac anatomy populations. Each sample shows the QRS complex for leads 1–8 (columns). (D) Per-lead MMD scores between real and virtual anatomy-based ECG populations (Proposed) and between two real anatomy-based ECG subpopulations (Gold Standard).

Due to the utility of accurate virtual 3D anatomy representations for many down-stream tasks, we investigate the ability of the Point VAE to generate realistic virtual heart populations based on the shape variability captured in its latent space. We therefore randomly sample 16-dimensional vectors from the multivariate latent space distribution and pass them through the pre-trained decoder of the Point VAE to generate synthetic biventricular point clouds (Fig. 5-A).

We observe that the generated virtual point clouds exhibit clear inter-subject shape variability that mimics the one found in true population. At the same time, each point cloud maintains a realistic appearance on an individual level, with relationships between different cardiac substructures retained in an anatomically plausible manner. Next, we quantify the realism of the virtual heart population as compared to the real one, based on multiple widely used clinical metrics for cardiac shape assessment. To this end, we draw 1000 samples from the Point VAE’s latent space distribution and reconstruct the corresponding point clouds. We calculate the LV volume, RV volume, and the LV mass for all anatomies of both the generated virtual population and the real population of the test dataset and report the obtained results for each population in Fig. 5-B. We find similar descriptive statistics (mean and standard deviation) between the virtual and gold standard populations.

Next, we investigate the utility of the generated virtual anatomies to act as inputs for *in silico* simulations of cardiac electrophysiology. To this end, we select 100 virtual point clouds and 100 real point clouds from the control group and apply multiple post-processing steps (see Section 2.7 for more details) to transform each heart into a suitable mesh representation for *in silico* simulations. Fig. 5-C depicts the resulting ECGs of two sample cases from the virtual and real anatomy population. When comparing the ECGs derived from the virtual and real anatomies, we observe similar types and levels of variability in QRS morphology across all 8 leads. For example, both populations feature QRS complexes with easily noticeable Q waves or S waves but also some where both waves are missing or considerably less pronounced. In order to quantify the similarity between the two generated ECG populations, we select the Maximum Mean Discrepancy (MMD) (Gretton et al., 2012) with a Gaussian kernel, since it is able to capture non-linear relationships and has been used in multiple prior works to compare ECG populations (Beetz et al., 2022a; Delaney et al., 2019). We calculate MMD scores between the real anatomy-based and virtual anatomy-based ECG populations separately for each lead and report the results in column 3 in Fig. 5-D. As a comparative benchmark, we randomly split the ECG population derived from real anatomies into two subsets and calculate the MMD between the resulting subpopulations (Fig. 5-D, column 2). We find that MMD scores between real and virtual anatomy-based ECG populations are lower than between two real anatomy-based ECG subpopulations for all 8 leads.

## 4 Discussion

We have presented the Point VAE as a novel geometric deep learning approach for 3D cardiac anatomy and function modeling as part of a fully automatic multi-step pipeline and evaluated its utility in multiple downstream tasks.

We have shown that the Point VAE is able to successfully extract important multi-scale shape features from complex 3D cardiac anatomies and achieve excellent reconstruction results with errors below the voxel resolution of the underlying image acquisition for multiple cardiac substructures at the ED and ES phases on a large dataset. This demonstrates its robustness to outliers and ability to successfully handle a variety of different non-linear spatio-temporal shape relations in a single unified model. It also indicates that the combined coarse and dense reconstruction architecture of the decoder branch and the update schedule of the weighting parameter *α* are suitably designed to achieve high local reconstruction quality without significantly affecting global consistency.

The resulting heart model takes population-wide variability in both cardiac anatomy and function into account at the same time, thus setting it apart from less holistic previous approaches based solely on one substructure or a single time point (Suinesiaputra et al., 2017; Beetz et al., 2021b, 2022d; Corral Acero et al., 2022). Furthermore, the Point VAE’s inference time after training is considerably below 1 s which enables real-time applications and scaling to large populations. The Point VAE does also not require any complex and time-consuming registration procedures and can thus be easily integrated into a fully automatic and fast 3D shape modeling pipeline with fewer prerequisites posed on the preceding surface reconstruction task. This increases the Point VAE’s flexibility as it can be applied to reconstructed 3D point cloud independently of the employed reconstruction method. The reconstruction step of the pipeline can therefore be tailored to the specific application and utilize either deep learning-based approaches as in this work (Beetz et al., 2023c) or more general reconstruction methods (Banerjee et al., 2021; Lamata et al., 2014) as needed.

In addition, the Point VAE’s latent space vector acts as a compressed representation of population variability in 3D cardiac anatomy and function with individual components responsible for encoding different aspects of shape variability. This shows the high degree of disentanglement of the latent space and improves the Point VAE’s applicability to downstream tasks and its interpretability as each component can be easily associated with 3D phenotypes. Furthermore, gradual value changes along individual components generally result in gradual changes in 3D shapes indicating that the latent space follows a smooth multivariate distribution. We also observed that different components encode different well-known aspects of 3D cardiac shape variability. This suggests that the selected latent space size of 16 is sufficiently large to enable the PointVAE to capture the key aspects of population-wide 3D shape variability while still retaining a compact and disentangled representation which is crucial for model interpretability. However, we hypothesize that other latent space sizes might also represent reasonable trade-offs depending on the specific clinical application as previously investigated for similar problems without a generative model setting (Beetz et al., 2023c).

Our experiments show that the Point VAE’s latent space representation can outperform multiple current clinical benchmarks by considerable margins for multiple MI prediction tasks. On the one hand, this demonstrates that pathology-specific 3D shape patterns in point clouds are adequately captured in the latent space and that a regression model can successfully utilize these novel 3D shape-based features for various downstream tasks. On the other hand, it shows the increased utility of relying on full 3D shape-based information coupled with efficient non-linear pattern extraction for the assessment of MI beyond single-valued volumetric biomarkers such as ejection fraction. The performance improvement is larger for prevalent MI detection than incident MI prediction, indicating that 3D information is especially beneficial for this task. We hypothesize this to be a result of post-infarction remodeling, which alters 3D cardiac shape. Furthermore, the outperformance of clinical benchmarks in MI survival analysis indicates that the Point VAE can extract more subtle features in order to succeed at the more difficult hazard prediction task. This is in addition to the coarser strictly binary prediction and is important for a more accurate stratification of patients at risk of MI events. We note, however, that the main goal of this work was not to achieve the best classification or survival prediction performance but rather to showcase the general utility and applicability of the Point VAE for this exemplary task.

The Point VAE’s excellent heart generation results show that its latent space distribution is of sufficient quality to successfully capture 3D shape variability across the population and that the decoder architecture is adequately designed to reconstruct virtual heart point clouds from random latent space samples with a high degree of accuracy. As such, virtual hearts still maintain a realistic appearance on an individual level, while at the same time exhibiting 3D shape variations similar to the real population. This is corroborated by the closeness in clinical metrics recorded for virtual and real heart populations in Fig. 5-B. We also observe that the generated heart populations can be successfully used as inputs to a cardiac electrophysiology simulation pipeline, which shows that the post-processing steps are adequately designed to fulfill the requirements of cardiac function modeling. Since these transformations can be executed in a fast and fully automatic manner, it opens up the possibility for larger scale *in silico* trials that take population-wide shape variability into account in greater detail but require fewer study-specific image acquisitions. This is important to further improve the accuracy and acceptance of *in silico* trials since variability in ventricular geometry has previously been shown to significantly affect ECG phenotypes (Mincholé et al., 2019). In addition, we find that the MMD scores between virtual and ground truth ECG populations are smaller than the scores between two ground truth ECG subpopulations for all 8 leads. Since all simulation-specific parameters were set to the same values when generating ECGs from real and virtual anatomies, all observed variations in ECG morphology are likely primarily the result of shape differences in the 3D anatomies. Consequently, the smaller MMD scores indicate that the 3D shape variability shows a higher resemblance between the virtual population and the total ground truth population than between two subsets of the ground population. This further corroborates the high realism of the virtual hearts generated by the Point VAE. The results are also consistent across all 8 leads, which shows that the virtual anatomies are suitable to model different aspects and phases of cardiac conductivity.

A limitation of the current simulation setup is that we did not change conduction velocities and root node locations, which has previously been shown to affect QRS morphology (Mincholé et al., 2019). Similarly, we have focused on generating only the QRS complex of a single cardiac cycle in healthy subjects. We note, however, that the primary aim of the experiment was to showcase the utility of the Point VAE’s generative ability for downstream simulation tasks under anatomically and physiologically plausible conditions as opposed to investigating the effect of different pathologies or post-processing parameter settings on QRS morphology or extending the simulation to the full ECG trace and multiple cardiac cycles. Nevertheless, we believe that the synthesized virtual anatomies have the potential to be used for such use cases.

In addition, we have only investigated 3D cardiac shape at two extreme ends of the cardiac cycle without taking the intermediate heart motion into account. Similarly, the anatomical representation has focused only on two of the hearts’ four chambers. However, both approximations are common in related research works and are often considered as the gold standard in clinical settings. Nevertheless, we believe that the presented approach can be extended to include additional cardiac substructures, such as the atria, and their variations along the full cardiac cycle. In general, all experiments conducted as part of this work are potentially affected by errors introduced during the pre-processing steps of the 3D heart dataset, including the MRI acquisition, image segmentation, and 3D reconstruction. Similarly, well-known biases and limitations of the UK Biobank study also apply here.

While the presented network architecture allowed the Point VAE to perform well in multiple tasks and constitutes, to the best of our knowledge, the first point cloud deep learning approach used to generate 3D heart models for cardiac electrophysiology simulations, it also comes with few disadvantages. First, the beta-VAE design requires considerable amounts of hyperparameter tuning which is time-consuming and data-dependent. Second, a larger number of network parameters is required for direct processing of point clouds as opposed to meshes due to their lack of connectivity information. Finally, more recent architectural developments, such as attention mechanisms, can be included in the presented Point VAE to further improve its performance. We plan to investigate such extensions in future work.

## 5 Conclusion

In this work, we have presented the Point VAE as a novel approach for 3D cardiac anatomy modeling and shown its suitability as the key component of a multi-step pipeline directly applicable to clinical gold standard cine MR images. We have found the Point VAE to be able to encode and reconstruct high-resolution multi-class point cloud representations of the cardiac anatomy with high degrees of accuracy while retaining a highly interpretable latent space representation. We have also presented that the case-specific latent encodings contain highly useful 3D shape information for myocardial infarction prediction, allowing them to considerably outperform multiple clinical benchmarks. Finally, we have demonstrated the Point VAE’s ability to generate realistic virtual populations of 3D hearts which exhibit similar characteristics as the ground truth population and are suitable to act as inputs for *in silico* simulations of cardiac electrophysiology.

## Figures and Tables

**Table 1:** Results of multiple regression models with various clinical biomarkers versus the Point VAE’s latent space as independent variables for the tasks of prevalent MI detection, incident MI prediction, and MI survival analysis.

Input	Prevalent MI						Incident MI					MI survival Harrell’s C-index
	Acc.	AUROC	F1	Prec.	Rec.		Acc.	AUROC	F1	Prec.	Rec.	
LV EF	0.600	0.662	0.568	0.621	0.537		0.581	0.636	0.544	0.601	0.515	0.5974
RV EF	0.583	0.618	0.556	0.601	0.534		0.512	0.558	0.477	0.532	0.467	0.5532
LV+RV EF	0.596	0.652	0.566	0.613	0.537		0.596	0.626	0.571	0.611	0.552	0.5928
Proposed	**0.710**	**0.768**	**0.780**	**0.737**	**0.833**		**0.655**	**0.717**	**0.648**	**0.659**	**0.648**	**0.6561**

Values represent the mean in all cases.

**Table 2:** Diseases and associated codes of UK Biobank field ID 20002 used in the definition of control cases.

Code	Meaning	Code	Meaning
1065	Hypertension	1286	Depression
1066	Heart/cardiac problem	1412	Bronchitis
1067	Peripheral vascular disease	1471	Atrial fibrillation
1072	Essential hypertension	1472	Emphysema
1073	Gestational hypertension/pre-eclampsia	1473	High cholesterol
1074	Angina	1483	Atrial flutter
1075	Heart attack/myocardial infarction	1484	Wolff Parkinson white/WPW syndrome
1076	Heart failure/pulmonary odema	1485	Irregular heart beat
1077	Heart arrhythmia	1486	Sick sinus syndrome
1078	Heart valve problem/heart murmur	1487	SVT/supraventricular tachycardia
1079	Cardiomyopathy	1491	Brain haemorrhage
1080	Pericardial problem	1492	Aortic aneurysm
1081	Stroke	1496	Alpha-1 antitrypsin deficiency
1086	Subarachnoid haemorrhage	1531	Post-natal depression
1087	Leg claudication/intermittent claudication	1583	Ischaemic stroke
1088	Arterial embolism	1584	Mitral valve disease
1111	Asthma	1585	Mitral regurgitation/incompetence
1112	Chronic obstructive airways disease/COPD	1586	Aortic valve disease
1113	Emphysema/chronic bronchitis	1587	Aortic regurgitation/incompetence
1220	Diabetes	1588	Hypertrophic cardiomyopathy
1221	Gestational diabetes	1589	Pericarditis
1222	Type 1 diabetes	1590	Pericardial effusion
1223	Type 2 diabetes	1591	Aortic aneurysm rupture
1262	Parkinson’s disease	1592	Aortic dissection
1263	Dementia/Alzheimer’s/cognitive impairment		

## Data Availability

Data will be made available on request.

## Appendix. Identification of control cases

Control cases were selected in this work to not suffer from any of the pathologies listed in Table 2.
